# Multinational survey shows low awareness of tick-borne encephalitis and rabies among travellers to endemic regions

**DOI:** 10.1093/jtm/tay069

**Published:** 2018-12-17

**Authors:** Cinzia Marano, Melissa Moodley, Elaine Melander, Laurence De Moerlooze, Hans D Nothdurft

**Affiliations:** 1 GSK, Wavre, Belgium; 2 Ipsos MORI, Ipsos Healthcare, London, UK; 3 Department of Infectious Diseases and Tropical Medicine, Medical Center of the University of Munich, Munich, Germany

In this supplement of the *Journal of Travel Medicine*, we describe travellers’ attitudes to rabies^1^ and tick-borne encephalitis (TBE)^2^ based on online panel surveys of adults who had travelled to a rabies or TBE endemic country or region in the past 3 years (visit-risk sample: 4678 for rabies and 4375 for TBE). For each disease, a sub-sample of travellers undertaking pre-defined activities that increased the risk of exposure to rabies (850) or TBE (375) received a more detailed questionnaire (activity-risk sub-samples). Healthcare providers (HCPs; 180) working at travel clinics were also interviewed using a telephone/online survey.

HCPs stated that typically, 60% of individuals visiting a travel clinic have some idea of the travel vaccinations needed, and about a third of travellers have done background research and are somewhat informed about which vaccines they need. HCPs also stated that a risk assessment conducted by a travel clinic typically involved questions about the type of trip, and medical history, and less commonly about the availability of medical care, organisation of trip, ability to pay for vaccine, insurance coverage, and whether the traveller is visiting friends and family. Travellers in the visit-risk samples reported seeking travel health information mostly online, followed by asking for information from doctors, pharmacists and healthcare centers, although there were some differences between the diseases. For rabies and TBE, respectively, 33% and 26% of travellers used online resources, and 24% and 8% consulted a family doctor.

The number of travellers stating that they sought information about rabies or TBE vaccinations was relatively low compared with a previous survey about hepatitis A and B vaccinations.^3^ The hepatitis survey was based on similar methodology as our study, and from a sample of travellers from seven countries, 70% sought advice from any HCP and 72% of respondents were aware of hepatitis. Indeed, in our surveys, when questioned about vaccines in general, the awareness of rabies and TBE vaccines was low (42% and 31%, respectively) compared with other vaccines against infectious diseases such as hepatitis A and B (70% and 72%, respectively). The high rate of advice-seeking for hepatitis A and B and higher awareness of hepatitis vaccines compared with rabies and TBE probably reflects a higher knowledge of the disease, given that even in most non-endemic countries such as the USA hepatitis A and B are recommended for routine childhood vaccination.^4,5^ However, although the global prevalence of reported human cases of rabies and TBE is much lower than that of hepatitis, exposure to these viruses can be deadly, and for travellers to endemic regions, represent a serious health risk.

In previous international surveys of travellers’ attitudes to travel vaccines, lack of awareness of the disease and the need for preventative measures were the most common reasons given for vaccine refusal.^6–12^ In the total visit-risk sample in our survey, a small proportion said they had a good knowledge of rabies (15%) and TBE (10%), and only 4% had never heard of rabies compared with 32% for TBE. However, awareness of a TBE vaccine was higher among travellers from the TBE endemic countries Germany (37%) and Sweden (70%) compared with travellers from non-endemic countries Canada (10%) and the UK (13%), whereas awareness of a rabies vaccine was similar in Germany (51%), Sweden (35%), Canada (42%) and the UK (40%). Moreover, the perceived risk of disease was low even among travellers in the activity-risk sub-samples; in unvaccinated travellers undertaking activities which increased the risk of exposure to rabies or TBE, 42% and 34%, respectively, did not think that the risk of disease was high enough to warrant vaccination.

In respective activity-risk sub-samples, the majority of travellers were aware of the need to wear long trousers and to avoid walking in long grass as a preventive measure for TBE, and awareness of the need to avoid wild animals to prevent rabies was high, but there was less certainty about rabies and domestic animals. Travellers from Germany, Sweden and the UK perceived the risk of rabies to be lower for visits to European countries (mainly Turkey, Poland and Croatia) compared with visits outside of the continent (mainly Mexico and Thailand), and this was also observed for Canadians who generally perceived the risk of rabies to be lower when travelling to Central America compared with Asia or Africa. A lower sense of risk of rabies is justified given that most of Europe and all of North America is dog rabies-free; however, wild animals in Europe, such as bats and foxes, can expose humans to rabies and represent a risk to travellers,^13,14^ particularly those visiting rural areas or spending time outdoors doing activities such as hiking and camping. Our study suggests that there is a need to raise awareness of the risk of rabies from wild animals in countries that are close to home where dog rabies is not a risk. Educating the travellers on post-exposure prophylaxis is also needed, as the majority were not aware of measures like washing the wound when bitten or scratched (53%) or receiving vaccination against rabies (60%).

Among HCPs, 61% stated that cost was a barrier to rabies vaccination among travellers, whereas in the rabies activity-risk sub-sample, just 14% of travellers said that the cost of vaccination discouraged them. In the TBE survey, both HCPs and travellers stated that a lack of perceived risk was the main reason to refuse TBE vaccination. If forced to prioritise and recommend just three vaccines to travellers on a low budget who are at risk of all conditions, HCPs said that they were most likely to select yellow fever, followed by hepatitis A and B, although this varied by country (Table 1). The relatively low priority observed for rabies and TBE vaccination compared with other travel vaccines against infectious diseases is consistent with previous reports. For example, in a large survey of travellers in Sweden presenting for pre-travel advice, the most commonly administered vaccine was hepatitis A, followed by yellow fever, whereas the uptake of rabies vaccine was relatively low, and TBE vaccine was not offered.^7^

**Table 1.** Travel clinic respondents prioritizing vaccines if only three can be given

	Total (%)	Canada (%)	Germany (%)	Sweden (%)	UK (%)
Yellow fever	62	66	74	67	42
Hepatitis A	57	66	52	88	32
Hepatitis B	56	45	40	88	62
Rabies	41	26	56	9	62
Typhoid	34	53	26	12	38
Meningitis	32	23	36	15	46
Cholera	19	21	16	21	18

Compliance with vaccine schedules most often relied on follow-up appointments and vaccination cards, and less often on e-mails, texts and phone calls, and for all travel vaccines provided, HCPs estimated that about 81% of travellers complete their vaccination course(s). However, in the activity-risk sub-samples, fewer than half of travellers who could remember the number of doses received for rabies or TBE vaccines stated that they received the full number of doses before travelling.

These findings suggest that the perceived risk of exposure to rabies or TBE among travellers to endemic regions underestimates the need to receive pre-travel vaccinations. Moreover, a third of travellers had never heard of TBE, and whereas rabies awareness was better, the perceived risk of exposure to rabies from wild animals in Europe was low. The most frequently cited source of pre-travel information was online, followed by consulting a HCP, suggesting that improved online resources about infectious diseases could increase awareness of the need for vaccinations among travellers.

## Supplementary Material

Supplementary Data
