# Supplementary figures and images for: Multinational survey shows low awareness of tick-borne encephalitis and rabies among travellers to endemic regions
Source: J Travel Med. 2018 Dec 17;26(Suppl 1):S1–2. doi: 10.1093/jtm/tay069 (PMC6376454; doi:10.1093/jtm/tay069)

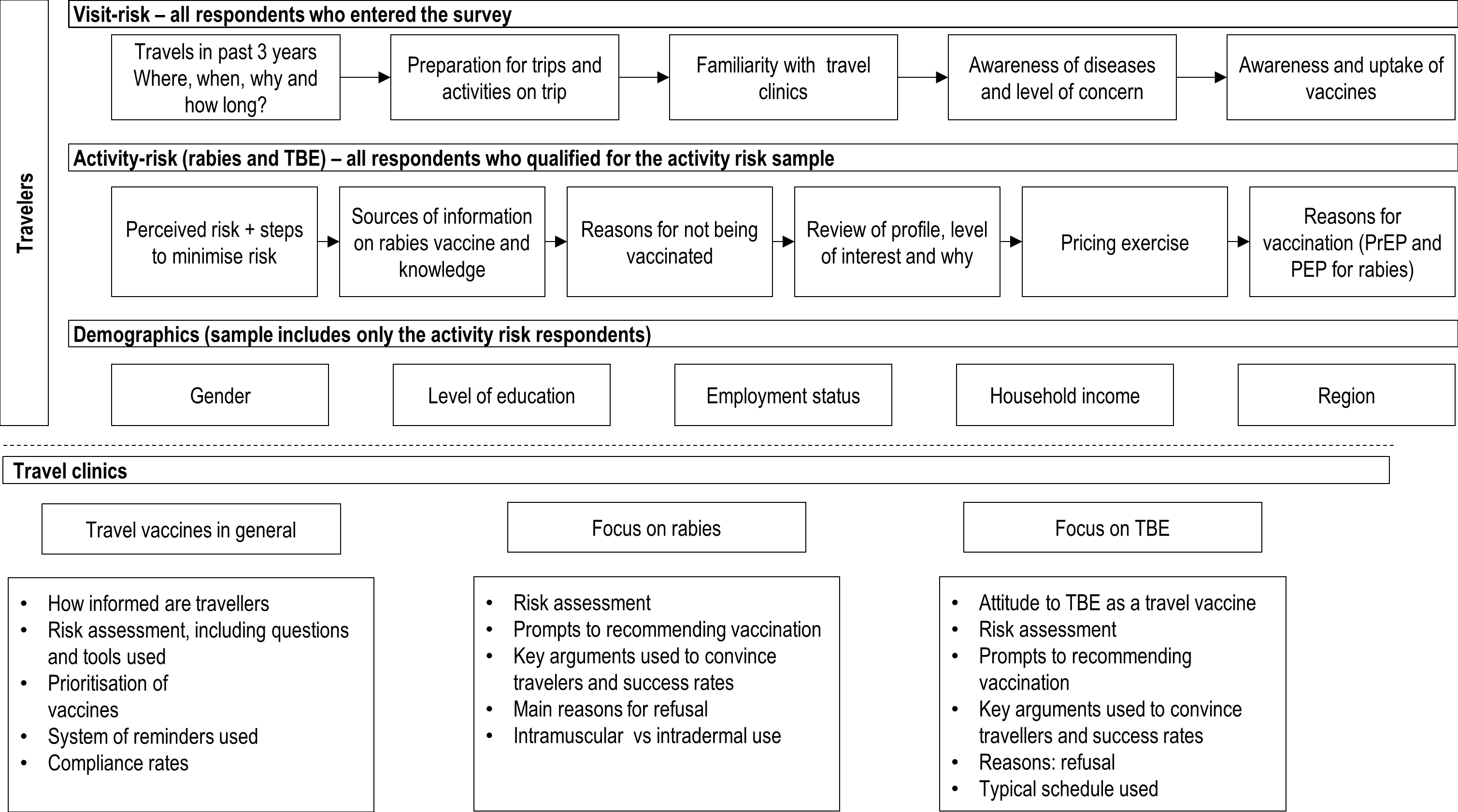

Supplement: Supplementary Data [file tay069_tram1501_supplementary_figure_a.png]
